# (*E*)-*N*′-[(*E*)-3-(4-Hydr­oxy-3-methoxy­phen­yl)allyl­idene]isonicotinohydrazide

**DOI:** 10.1107/S1600536810000371

**Published:** 2010-01-09

**Authors:** H. S. Naveenkumar, Amirin Sadikun, Pazilah Ibrahim, Ching Kheng Quah, Hoong-Kun Fun

**Affiliations:** a School of Pharmaceutical Sciences, Universiti Sains Malaysia, 11800 USM, Penang, Malaysia; b X-ray Crystallography Unit, School of Physics, Universiti Sains Malaysia, 11800 USM, Penang, Malaysia

## Abstract

In the title compound, C_16_H_15_N_3_O_3_, the dihedral angle between the pyridine and benzene rings is 7.66 (5)°. The crystal packing is consolidated by inter­molecular C—H⋯O and O—H⋯N inter­actions, which link the mol­ecules into zigzag chains propagating along [010]. The chains are further linked into a three-dimensional network by N—H⋯O, C—H⋯N, C—H⋯O and C—H⋯π inter­actions.

## Related literature

For the synthesis, see: Lourenco *et al.* (2008 ▶). For the tuberculostatic activities of isoniazid derivatives, see: Janin (2007 ▶). For related structures, see: Naveenkumar *et al.* (2009*a* ▶,*b* ▶,*c* ▶); Shi (2005 ▶). For the stability of the temperature controller used for the data collection, see: Cosier & Glazer (1986 ▶).
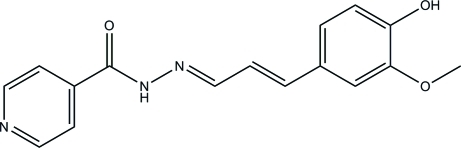


## Experimental

### 

#### Crystal data

C_16_H_15_N_3_O_3_
*M* *_r_* = 297.31
Monoclinic, 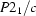

*a* = 5.0470 (1) Å
*b* = 28.9314 (6) Å
*c* = 9.6446 (2) Å
β = 90.010 (1)°
*V* = 1408.27 (5) Å^3^
*Z* = 4
Mo *K*α radiation
μ = 0.10 mm^−1^
*T* = 100 K
0.53 × 0.20 × 0.08 mm

#### Data collection

Bruker SMART APEXII CCD diffractometer
Absorption correction: multi-scan (*SADABS*; Bruker, 2009 ▶) *T*_min_ = 0.950, *T*_max_ = 0.992
33857 measured reflections
4144 independent reflections
3534 reflections with *I* > 2σ(*I*)
*R*_int_ = 0.030

#### Refinement

*R*[*F* ^2^ > 2σ(*F* ^2^)] = 0.043
*wR*(*F* ^2^) = 0.110
*S* = 1.05
4144 reflections
208 parameters
H atoms treated by a mixture of independent and constrained refinement
Δρ_max_ = 0.43 e Å^−3^
Δρ_min_ = −0.22 e Å^−3^

### 

Data collection: *APEX2* (Bruker, 2009 ▶); cell refinement: *SAINT* (Bruker, 2009 ▶); data reduction: *SAINT*; program(s) used to solve structure: *SHELXTL* (Sheldrick, 2008 ▶); program(s) used to refine structure: *SHELXTL*; molecular graphics: *SHELXTL*; software used to prepare material for publication: *SHELXTL* and *PLATON* (Spek, 2009 ▶).

## Supplementary Material

Crystal structure: contains datablocks global, I. DOI: 10.1107/S1600536810000371/hb5304sup1.cif

Structure factors: contains datablocks I. DOI: 10.1107/S1600536810000371/hb5304Isup2.hkl

Additional supplementary materials:  crystallographic information; 3D view; checkCIF report

## Figures and Tables

**Table 1:** Hydrogen-bond geometry (Å, °) *Cg*1 is the centroid of the C10–C15 benzene ring.

*D*—H⋯*A*	*D*—H	H⋯*A*	*D*⋯*A*	*D*—H⋯*A*
N2—H1*N*2⋯O1^i^	0.886 (15)	1.999 (15)	2.8622 (12)	164.4 (14)
O2—H1*O*2⋯N1^ii^	0.87 (2)	1.96 (2)	2.7750 (13)	156.9 (19)
C2—H2*A*⋯O3^iii^	0.93	2.55	3.1651 (14)	124
C4—H4*A*⋯N3^iv^	0.93	2.60	3.4747 (14)	156
C7—H7*A*⋯O1^i^	0.93	2.52	3.2405 (14)	135
C16—H16*B*⋯*Cg*1^v^	0.96	2.65	3.4556 (12)	142

Symmetry codes: (i) 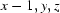; (ii) 
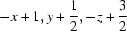
; (iii) 
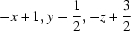
; (iv) 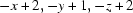; (v) 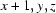.

**Fig. 1.**
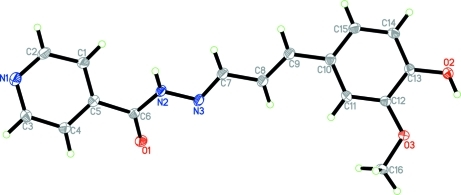
The asymmetric unit of (I) showing 50% displacement ellipsoids.

**Fig. 2.**
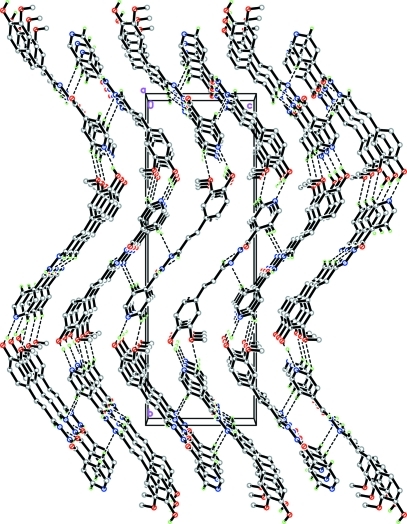
The crystal packing of (I), viewed down the a axis. H atoms not involving in hydrogen bonds (dashed lines) have been omitted for clarity.

## supplementary crystallographic information

### Comment

In the search of new compounds, isoniazid derivatives have been found to possess potential tuberculostatic activities (e.g. Janin, 2007). As a part of a current work of synthesis of (E)-N'-substituted isonicotinohydrazide derivatives, we present the crystal structure of the title compound, (I), (Fig. 1).

The pyridine ring (N1/C1–C5) in (I) forms dihedral angle of 7.66 (5)° with the benzene ring (C10–C15), indicating that they are almost co-planar to each other. Bond lengths and angles are within normal ranges, and comparable to closely related structures (Naveenkumar *et al.*, 2009a,b,c; Shi, 2005).

The crystal packing is consolidated by intermolecular C2—H2A···O3 and O2—H1O2···N1 interactions (Fig. 2) which link the independent molecules into zig-zag chains along the [0 1 0] direction. The crystal structure is further linked *via* N2—H1N2···O1, C4—H4A···N3 and C7—H7A···O1 interactions, into three-dimensional network. The structure is also stabilized by C—H···π interactions (Table 1).

### Experimental

The isoniazid derivative was prepared following the procedure by Lourenco *et al.* (2008). (E)-N'-[(E)-3-(4-hydroxy-3-methoxyphenyl) allylidene]isonicotinohydrazide was prepared by reaction between the 4-hydroxy-3-methoxy cinnamaldehyde (1.0 eq) with isoniazid (1.0 eq) in ethanol/water. After stirring for 1-3 h at room temperature, the resulting mixture was concentrated under reduced pressure. The residue, purified by washing with cold ethanol and ethyl ether, afforded the pure derivative. Yellow needles of (I) were obtained by recrystallization from  methanol.

### Refinement

Atoms H1N2 and H1O2 were located in a difference Fourier map and refined freely. The remaining H atoms were positioned geometrically and refined using a riding model, with C-H = 0.93–0.96 Å and *U*_iso_(H) = 1.2 or 1.5 *U*_eq_(C). A rotating-group model was applied for the methyl groups.

### Crystal data

**Table:**

C_16_H_15_N_3_O_3_	*F*(000) = 624
*M**_r_* = 297.31	*D*_x_ = 1.402 Mg m^−^^3^
Monoclinic, *P*2_1_/*c*	Mo *K*α radiation, λ = 0.71073 Å
Hall symbol: -P 2ybc	Cell parameters from 9970 reflections
*a* = 5.0470 (1) Å	θ = 2.2–30.1°
*b* = 28.9314 (6) Å	µ = 0.10 mm^−^^1^
*c* = 9.6446 (2) Å	*T* = 100 K
β = 90.010 (1)°	Needle, yellow
*V* = 1408.27 (5) Å^3^	0.53 × 0.20 × 0.08 mm
*Z* = 4	

### Data collection

**Table:**

Bruker SMART APEXII CCD diffractometer	4144 independent reflections
Radiation source: fine-focus sealed tube	3534 reflections with *I* > 2σ(*I*)
graphite	*R*_int_ = 0.030
φ and ω scans	θ_max_ = 30.2°, θ_min_ = 1.4°
Absorption correction: multi-scan (*SADABS*; Bruker, 2009)	*h* = −7→7
*T*_min_ = 0.950, *T*_max_ = 0.992	*k* = −40→40
33857 measured reflections	*l* = −13→13

### Refinement

**Table:**

Refinement on *F*^2^	Primary atom site location: structure-invariant direct methods
Least-squares matrix: full	Secondary atom site location: difference Fourier map
*R*[*F*^2^ > 2σ(*F*^2^)] = 0.043	Hydrogen site location: inferred from neighbouring sites
*wR*(*F*^2^) = 0.110	H atoms treated by a mixture of independent and constrained refinement
*S* = 1.05	*w* = 1/[σ^2^(*F*_o_^2^) + (0.0505*P*)^2^ + 0.5887*P*] where *P* = (*F*_o_^2^ + 2*F*_c_^2^)/3
4144 reflections	(Δ/σ)_max_ = 0.001
208 parameters	Δρ_max_ = 0.43 e Å^−^^3^
0 restraints	Δρ_min_ = −0.22 e Å^−^^3^

### Special details

**Table:**

Experimental. The crystal was placed in the cold stream of an Oxford Cyrosystems Cobra open-flow nitrogen cryostat (Cosier & Glazer, 1986) operating at 100.0 (1) K.
Geometry. All esds (except the esd in the dihedral angle between two l.s. planes) are estimated using the full covariance matrix. The cell esds are taken into account individually in the estimation of esds in distances, angles and torsion angles; correlations between esds in cell parameters are only used when they are defined by crystal symmetry. An approximate (isotropic) treatment of cell esds is used for estimating esds involving l.s. planes.
Refinement. Refinement of F^2^ against ALL reflections. The weighted R-factor wR and goodness of fit S are based on F^2^, conventional R-factors R are based on F, with F set to zero for negative F^2^. The threshold expression of F^2^ > 2sigma(F^2^) is used only for calculating R-factors(gt) etc. and is not relevant to the choice of reflections for refinement. R-factors based on F^2^ are statistically about twice as large as those based on F, and R- factors based on ALL data will be even larger.

### Fractional atomic coordinates and isotropic or equivalent isotropic displacement parameters (Å^2^)

**Table:**

	*x*	*y*	*z*	*U*_iso_*/*U*_eq_	
O1	1.08233 (15)	0.46282 (3)	0.88942 (9)	0.01916 (18)	
O2	0.21767 (17)	0.74390 (3)	0.22611 (9)	0.01855 (18)	
O3	0.60188 (15)	0.73516 (3)	0.41721 (8)	0.01721 (17)	
N1	0.7016 (2)	0.32923 (3)	1.14947 (11)	0.0203 (2)	
N2	0.64104 (19)	0.47193 (3)	0.84655 (10)	0.01645 (19)	
N3	0.68169 (19)	0.51003 (3)	0.76213 (10)	0.0175 (2)	
C1	0.5730 (2)	0.38057 (4)	0.96577 (15)	0.0242 (3)	
H1A	0.4555	0.3873	0.8943	0.029*	
C2	0.5420 (2)	0.34088 (4)	1.04462 (15)	0.0260 (3)	
H2A	0.4020	0.3212	1.0233	0.031*	
C3	0.8987 (3)	0.35853 (4)	1.17893 (13)	0.0235 (3)	
H3A	1.0093	0.3515	1.2531	0.028*	
C4	0.9479 (2)	0.39885 (4)	1.10550 (12)	0.0213 (2)	
H4A	1.0887	0.4180	1.1294	0.026*	
C5	0.7823 (2)	0.41004 (4)	0.99545 (11)	0.0149 (2)	
C6	0.8505 (2)	0.45104 (4)	0.90682 (11)	0.0144 (2)	
C7	0.4745 (2)	0.52317 (4)	0.69452 (12)	0.0179 (2)	
H7A	0.3181	0.5063	0.7002	0.021*	
C8	0.4869 (2)	0.56419 (4)	0.61002 (12)	0.0184 (2)	
H8A	0.6425	0.5814	0.6096	0.022*	
C9	0.2819 (2)	0.57851 (4)	0.53159 (12)	0.0181 (2)	
H9A	0.1349	0.5592	0.5273	0.022*	
C10	0.2689 (2)	0.62152 (4)	0.45276 (11)	0.0158 (2)	
C11	0.4478 (2)	0.65790 (4)	0.47779 (11)	0.0158 (2)	
H11A	0.5762	0.6546	0.5463	0.019*	
C12	0.4357 (2)	0.69838 (4)	0.40220 (11)	0.0141 (2)	
C13	0.2379 (2)	0.70419 (4)	0.29982 (11)	0.0144 (2)	
C14	0.0603 (2)	0.66845 (4)	0.27564 (12)	0.0173 (2)	
H14A	−0.0695	0.6719	0.2080	0.021*	
C15	0.0737 (2)	0.62759 (4)	0.35120 (12)	0.0173 (2)	
H15A	−0.0478	0.6041	0.3342	0.021*	
C16	0.7875 (2)	0.73391 (4)	0.52991 (12)	0.0176 (2)	
H16A	0.9059	0.7598	0.5229	0.026*	
H16B	0.8873	0.7057	0.5259	0.026*	
H16C	0.6934	0.7354	0.6163	0.026*	
H1N2	0.476 (3)	0.4640 (5)	0.8667 (16)	0.025 (4)*	
H1O2	0.284 (4)	0.7669 (7)	0.272 (2)	0.048 (6)*	

### Atomic displacement parameters (Å^2^)

**Table:**

	*U*^11^	*U*^22^	*U*^33^	*U*^12^	*U*^13^	*U*^23^
O1	0.0127 (4)	0.0203 (4)	0.0245 (4)	−0.0018 (3)	0.0008 (3)	0.0028 (3)
O2	0.0221 (4)	0.0158 (4)	0.0178 (4)	−0.0010 (3)	−0.0061 (3)	0.0024 (3)
O3	0.0162 (4)	0.0151 (4)	0.0203 (4)	−0.0022 (3)	−0.0061 (3)	0.0015 (3)
N1	0.0241 (5)	0.0162 (5)	0.0204 (5)	−0.0010 (4)	0.0014 (4)	0.0014 (4)
N2	0.0127 (4)	0.0140 (4)	0.0226 (5)	−0.0011 (3)	0.0009 (3)	0.0045 (4)
N3	0.0172 (4)	0.0131 (4)	0.0221 (5)	−0.0014 (3)	0.0008 (3)	0.0035 (4)
C1	0.0188 (5)	0.0180 (6)	0.0358 (7)	−0.0030 (4)	−0.0089 (5)	0.0068 (5)
C2	0.0201 (6)	0.0178 (6)	0.0400 (7)	−0.0052 (4)	−0.0061 (5)	0.0068 (5)
C3	0.0312 (6)	0.0218 (6)	0.0175 (5)	−0.0057 (5)	−0.0059 (4)	0.0030 (5)
C4	0.0249 (6)	0.0206 (6)	0.0183 (5)	−0.0066 (4)	−0.0042 (4)	0.0012 (4)
C5	0.0139 (5)	0.0130 (5)	0.0178 (5)	0.0010 (4)	0.0029 (4)	−0.0013 (4)
C6	0.0139 (5)	0.0128 (5)	0.0163 (5)	−0.0004 (4)	0.0009 (4)	−0.0009 (4)
C7	0.0162 (5)	0.0154 (5)	0.0220 (5)	−0.0018 (4)	0.0009 (4)	0.0018 (4)
C8	0.0169 (5)	0.0154 (5)	0.0228 (6)	−0.0009 (4)	0.0011 (4)	0.0025 (4)
C9	0.0170 (5)	0.0153 (5)	0.0221 (6)	−0.0014 (4)	0.0006 (4)	0.0018 (4)
C10	0.0154 (5)	0.0155 (5)	0.0166 (5)	0.0012 (4)	0.0008 (4)	0.0006 (4)
C11	0.0136 (5)	0.0168 (5)	0.0171 (5)	0.0015 (4)	−0.0021 (4)	0.0010 (4)
C12	0.0124 (4)	0.0149 (5)	0.0149 (5)	0.0004 (4)	0.0003 (3)	−0.0018 (4)
C13	0.0157 (5)	0.0152 (5)	0.0122 (5)	0.0018 (4)	−0.0003 (3)	−0.0003 (4)
C14	0.0178 (5)	0.0186 (5)	0.0155 (5)	−0.0005 (4)	−0.0042 (4)	−0.0015 (4)
C15	0.0166 (5)	0.0164 (5)	0.0190 (5)	−0.0018 (4)	−0.0013 (4)	−0.0022 (4)
C16	0.0154 (5)	0.0197 (5)	0.0178 (5)	−0.0003 (4)	−0.0045 (4)	−0.0015 (4)

### Geometric parameters (Å, °)

**Table:**

O1—C6	1.2301 (13)	C5—C6	1.5021 (15)
O2—C13	1.3550 (13)	C7—C8	1.4410 (16)
O2—H1O2	0.87 (2)	C7—H7A	0.9300
O3—C12	1.3626 (13)	C8—C9	1.3470 (16)
O3—C16	1.4352 (13)	C8—H8A	0.9300
N1—C2	1.3360 (16)	C9—C10	1.4598 (15)
N1—C3	1.3373 (16)	C9—H9A	0.9300
N2—C6	1.3494 (14)	C10—C15	1.4000 (15)
N2—N3	1.3856 (13)	C10—C11	1.4075 (15)
N2—H1N2	0.884 (17)	C11—C12	1.3808 (15)
N3—C7	1.2895 (15)	C11—H11A	0.9300
C1—C2	1.3862 (17)	C12—C13	1.4142 (15)
C1—C5	1.3875 (16)	C13—C14	1.3881 (15)
C1—H1A	0.9300	C14—C15	1.3904 (16)
C2—H2A	0.9300	C14—H14A	0.9300
C3—C4	1.3871 (17)	C15—H15A	0.9300
C3—H3A	0.9300	C16—H16A	0.9600
C4—C5	1.3889 (16)	C16—H16B	0.9600
C4—H4A	0.9300	C16—H16C	0.9600
			
C13—O2—H1O2	110.8 (13)	C9—C8—H8A	118.7
C12—O3—C16	117.55 (9)	C7—C8—H8A	118.7
C2—N1—C3	116.70 (10)	C8—C9—C10	126.10 (10)
C6—N2—N3	119.56 (9)	C8—C9—H9A	117.0
C6—N2—H1N2	121.8 (10)	C10—C9—H9A	117.0
N3—N2—H1N2	118.3 (10)	C15—C10—C11	118.52 (10)
C7—N3—N2	114.31 (9)	C15—C10—C9	120.18 (10)
C2—C1—C5	118.81 (11)	C11—C10—C9	121.29 (10)
C2—C1—H1A	120.6	C12—C11—C10	121.02 (10)
C5—C1—H1A	120.6	C12—C11—H11A	119.5
N1—C2—C1	123.79 (11)	C10—C11—H11A	119.5
N1—C2—H2A	118.1	O3—C12—C11	125.39 (10)
C1—C2—H2A	118.1	O3—C12—C13	114.57 (9)
N1—C3—C4	123.91 (11)	C11—C12—C13	120.04 (10)
N1—C3—H3A	118.0	O2—C13—C14	119.67 (10)
C4—C3—H3A	118.0	O2—C13—C12	121.34 (10)
C3—C4—C5	118.58 (11)	C14—C13—C12	118.99 (10)
C3—C4—H4A	120.7	C13—C14—C15	120.91 (10)
C5—C4—H4A	120.7	C13—C14—H14A	119.5
C1—C5—C4	118.19 (11)	C15—C14—H14A	119.5
C1—C5—C6	122.84 (10)	C14—C15—C10	120.51 (10)
C4—C5—C6	118.76 (10)	C14—C15—H15A	119.7
O1—C6—N2	124.21 (10)	C10—C15—H15A	119.7
O1—C6—C5	120.96 (10)	O3—C16—H16A	109.5
N2—C6—C5	114.79 (9)	O3—C16—H16B	109.5
N3—C7—C8	119.57 (10)	H16A—C16—H16B	109.5
N3—C7—H7A	120.2	O3—C16—H16C	109.5
C8—C7—H7A	120.2	H16A—C16—H16C	109.5
C9—C8—C7	122.51 (10)	H16B—C16—H16C	109.5
			
C6—N2—N3—C7	170.58 (10)	C8—C9—C10—C15	165.29 (12)
C3—N1—C2—C1	1.0 (2)	C8—C9—C10—C11	−15.62 (18)
C5—C1—C2—N1	0.6 (2)	C15—C10—C11—C12	−1.36 (16)
C2—N1—C3—C4	−1.61 (19)	C9—C10—C11—C12	179.53 (10)
N1—C3—C4—C5	0.7 (2)	C16—O3—C12—C11	−6.79 (15)
C2—C1—C5—C4	−1.55 (18)	C16—O3—C12—C13	172.87 (9)
C2—C1—C5—C6	173.17 (11)	C10—C11—C12—O3	−178.97 (10)
C3—C4—C5—C1	0.97 (18)	C10—C11—C12—C13	1.38 (16)
C3—C4—C5—C6	−173.96 (11)	O3—C12—C13—O2	−1.11 (15)
N3—N2—C6—O1	−1.93 (17)	C11—C12—C13—O2	178.58 (10)
N3—N2—C6—C5	−179.76 (9)	O3—C12—C13—C14	179.36 (10)
C1—C5—C6—O1	−144.63 (12)	C11—C12—C13—C14	−0.95 (16)
C4—C5—C6—O1	30.05 (16)	O2—C13—C14—C15	−179.00 (10)
C1—C5—C6—N2	33.28 (15)	C12—C13—C14—C15	0.54 (16)
C4—C5—C6—N2	−152.04 (11)	C13—C14—C15—C10	−0.54 (17)
N2—N3—C7—C8	176.04 (10)	C11—C10—C15—C14	0.93 (16)
N3—C7—C8—C9	176.67 (11)	C9—C10—C15—C14	−179.95 (10)
C7—C8—C9—C10	174.30 (11)		

### Hydrogen-bond geometry (Å, °)

**Table:**

Cg1 is the centroid of the C10–C15 benzene ring.

**Table:**

*D*—H···*A*	*D*—H	H···*A*	*D*···*A*	*D*—H···*A*
N2—H1N2···O1^i^	0.886 (15)	1.999 (15)	2.8622 (12)	164.4 (14)
O2—H1O2···N1^ii^	0.87 (2)	1.96 (2)	2.7750 (13)	156.9 (19)
C2—H2A···O3^iii^	0.93	2.55	3.1651 (14)	124
C4—H4A···N3^iv^	0.93	2.60	3.4747 (14)	156
C7—H7A···O1^i^	0.93	2.52	3.2405 (14)	135
C16—H16B···Cg1^v^	0.96	2.65	3.4556 (12)	142

Symmetry codes: (i) *x*−1, *y*, *z*; (ii) −*x*+1, *y*+1/2, −*z*+3/2; (iii) −*x*+1, *y*−1/2, −*z*+3/2; (iv) −*x*+2, −*y*+1, −*z*+2; (v) *x*+1, *y*, *z*.
